# Suppression of Systematic Errors of Electronic Distance Meters for Measurement of Short Distances

**DOI:** 10.3390/s150819264

**Published:** 2015-08-06

**Authors:** Jaroslav Braun, Martin Štroner, Rudolf Urban, Filip Dvořáček

**Affiliations:** Department of Special Geodesy, Czech Technical University in Prague, Faculty of Civil Engineering, Thákurova 7, 166 29 Prague 6, Czech Republic; E-Mails: martin.stroner@fsv.cvut.cz (M.Š.); rudolf.urban@fsv.cvut.cz (R.U.); filip.dvoracek@fsv.cvut.cz (F.D.)

**Keywords:** EDM, baseline, interferometer, systematic error, calibration, short distance, standard deviation, pillar, forced centring, absolute distance

## Abstract

In modern industrial geodesy, high demands are placed on the final accuracy, with expectations currently falling below 1 mm. The measurement methodology and surveying instruments used have to be adjusted to meet these stringent requirements, especially the total stations as the most often used instruments. A standard deviation of the measured distance is the accuracy parameter, commonly between 1 and 2 mm. This parameter is often discussed in conjunction with the determination of the real accuracy of measurements at very short distances (5–50 m) because it is generally known that this accuracy cannot be increased by simply repeating the measurement because a considerable part of the error is systematic. This article describes the detailed testing of electronic distance meters to determine the absolute size of their systematic errors, their stability over time, their repeatability and the real accuracy of their distance measurement. Twenty instruments (total stations) have been tested, and more than 60,000 distances in total were measured to determine the accuracy and precision parameters of the distance meters. Based on the experiments’ results, calibration procedures were designed, including a special correction function for each instrument, whose usage reduces the standard deviation of the measurement of distance by at least 50%.

## 1. Introduction

Electronic distance meters (EDM) have an important role to play in modern land surveying. Most geodetic tasks are based on the direct measurement of distance, and the accuracy of the results is directly dependent on their functioning correctly and on the EDM precision. Distance meters have developed considerably since their creation in the 1940s and have become miniature components of total stations [1]. Current distance meters’ accuracies are typically from 1 to 2 mm. The standard deviation of the determined distance *D* is commonly presented by manufacturers as follows:

(1) $${\text{σ}}_{D}=A+B\cdot D$$

According to [2], an A value is given in millimeters and includes the reading accuracy of the EDM, the maximum amplitude (or average size) of the short cyclic error for the phase distance in meters, the maximum (or average) nonlinear effect of the distance-dependent errors and the accuracy of the additive constant. Value B is given in ppm (parts per million) and the distance D is given in kilometers. The B value for a short-range distance meter includes master frequency oscillator drift in the working temperature range and the maximum error that can be caused by limiting the steps in the calculation. This list is a mix of systematic and random errors. In addition to these, phenomena affecting the accuracy of the distance measurement also include the surveyor (operator), the current state of the environment (atmosphere) and any additional equipment used.

A summary of all of these phenomena leads to an error model ([3]):

(2) $$errorbudget=\sum \left(systematicerrors\right)+\sum \left(randomerrors\right)+noise$$

By appropriately adjusting the measurement procedures, it is possible to achieve better results. The effects of random errors can be suppressed by increasing the number of repetitions of the measurement. Based on general knowledge on EDM, and long-term experience of the authors, a suitable calibration procedure can determine the systematic errors and appropriate corrections can suppress their influence.

Routine tests mainly focus on the error of determining the additive prism constant [4], scaling depending on the distance [5], the time element error for pulsed distance meters [6], and the cyclic error of phase distance meters [7]. Standard ISO 17123-4 [8] (replacing ISO 8322-8 [9]), which defines the field tests of the distance meters to verify the accuracy and determine the additive prism constant, exists for the testing of the EDM. Calibration baselines are constructed at the level of national standards and following the international standards [10,11]. The main area on which the calibration focuses are long distances [12]. For calibration, outdoor pillar baselines of lengths typically between 800 and 1500 m are set up. Distances between forced centerings on the pillars are typically determined with a precision from 0.5 to 4.0 mm (in case of the new determinations up to 0.3 ppm·D or less) [13,14]. The absolute sizes of the errors are determined at the pillar bases as the difference between the length and the directly measured distance [15,16].

The calibration of total stations used in engineering surveying sometimes uses laboratory baselines with distance simultaneously determined by an interferometer, but the determination of the absolute errors there is much more difficult and, therefore, they are instead determined by the relative sizes of the errors (the differences between the relative distances measured by the interferometer and measured by the total station). The laboratory base is usually from 20 to 50 m long and typically consists of a rail for placement and movement of the prism and the interferometer; A standard deviation of the distance measured by the interferometer is approximately 1.5 ppm·D [17,18]. Sizes of deviations from the reference lengths are a standard output of the EDM calibrations. These deviations are often fitted by a line; its parameters represent the residual error of the additive constant and the error dependent on the distance. The deviations can also be interspersed with periodic functions by using the Fourier Transform [19].

In precise industrial engineering surveying the measurement of distance is usually in the range of 5–100 m. The above-described calibration procedures may not meet the required accuracy requirements, and corrections determined from long baselines may not represent real corrections for very short distances. There is also a hypothesis that the development of the additive constant varies significantly in the first 10 m [3] and that the distance meters measure short distances more precisely than the manufacturers of total stations claim.

Due to these hypotheses and for the reasons set out above, the Department of Special Geodesy, Faculty of Civil Engineering Czech Technical University in Prague designed an experimental EDM calibration procedure for determining the size of systematic errors and mechanisms for their suppression, to achieve more accurate measurement results. The procedure was designed for very precise measurements in industry, at short distances (up to 40 m) in closed factory buildings where stable atmospheric conditions can be assumed. A new laboratory pillar baseline with forced centerings with absolute lengths determined with a precision of 0.02 mm at the Faculty of Civil Engineering in Prague (Czech Republic) and a laboratory baseline with an interferometer at the Department of Geodesy TU Dresden (Germany) were used for the experiment. Eight different types of distance meters (20 instruments in total) were tested. Three selected instruments were fully tested and verification measurements were performed to test the relevance of the proposed calibration procedure. The paper addresses commonly available equipment and does not deal with high-precision specialized equipment with high accuracy, such as ME Kern 5000 [20], *etc.*

The designed procedure aims to acquire more precise and accurate results of distance measurement using EDM by suppression of systematic errors by special corrections and random errors by modification of the measurement procedure.

## 2. Experimental Section

The proposed experimental procedure for determining of the sizes of systematic errors at short distances and correcting directly measured distances by the EDM consists of nine successive steps.

(a) Selection of the instrument, which is traceable to the length standard, to determine the absolute distances.
(b) Construction of the laboratory pillar baseline.
(c) Choice and testing of the equipment for laboratory measurements on the baseline (tribrachs, reflectors, *etc.*).
(d) Determination of the absolute laboratory baseline lengths.
(e) Determination of the size of the absolute errors of selected distance meters and their stability over time.
(f) Test of the different distance meters of the same type to compare the size of the absolute errors.
(g) Determination of relative errors of three distance meters in detail on the baseline with an interferometer.
(h) Determination of the correction function for three distance meters, based on a combination of absolute and relative errors.
(i) Experimental verification of the suitability and quality of the measured distances’ corrections.

### 2.1. Reference Instrument

**Figure 1:**
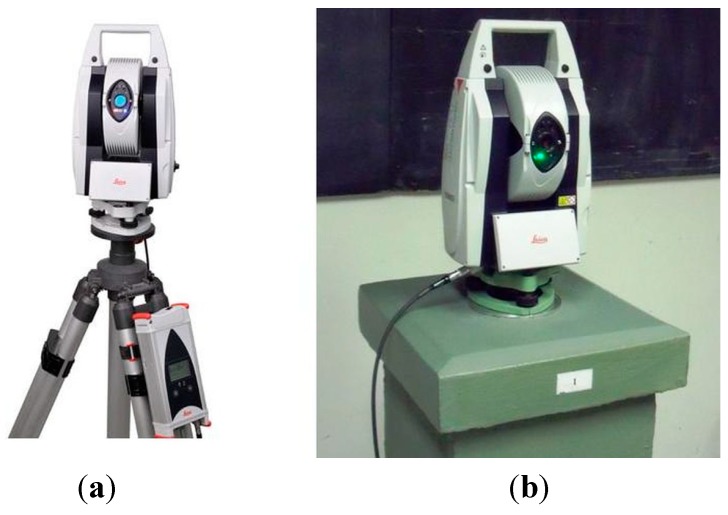
Leica absolute tracker AT401: (**a**) the tracker at a tripod; (**b**) the tracker at the pillar no. 1 in laboratory.

Distance meters in total stations commonly determine the distance with a resolution of 0.1 mm; it was therefore necessary to choose a reference instrument that could determine the reference distance to an absolute accuracy of better than 0.05 mm. Another condition was a metrological connection to the national length standard. Therefore, the Leica Absolute Tracker AT401 (Figure 1) was selected; the instrument has been loaned by the Czech Research Institute of Geodesy, Topography and Cartography (VÚGTK), which is a government agency charged with maintaining Czech calibration bases and national standards of long lengths and with issuing certificates of calibration for surveying instruments in the Czech Republic [21].

The Leica Absolute Tracker AT401 [22] is primarily designed for very precise engineering measurements. The accuracy of the angular measurement is characterized by the standard deviation σ_φ_ = 0.15 mgon (resolution 0.02 mgon). The accuracy of the distance measurement is characterized by the standard deviation σ_D_ = 5 μm (resolution 0.1 µm). The maximum range of the distance measurement is 160 m. For accurate results, appropriate knowledge of the physical properties of the air (temperature, pressure, and relative humidity) is required; these parameters are measured by external sensors.

**Figure 2:**
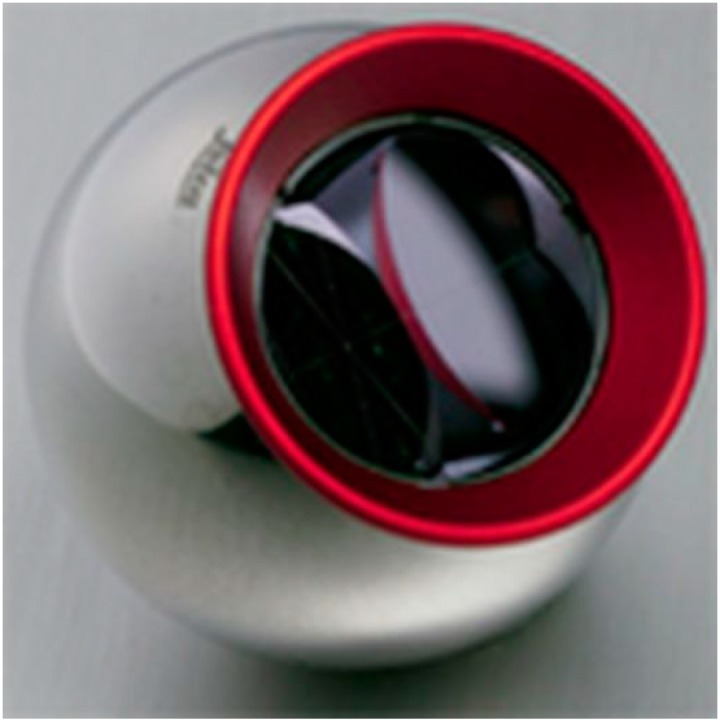
Leica Red-Ring Reflector 1.5″ (RRR) with removable ring.

A spherical prism, Leica RRR 1.5″ (Figure 2), is used as a target. It is characterized by the shape accuracy of 0.0025 mm and accuracy of fit of reflective surfaces of 0.003 mm. The additive constant is set to be 0.00 mm; its actual size was determined by a procedure provided by the instrument manufacturer.

### 2.2. Laboratory Pillar Baseline

**Figure 3:**
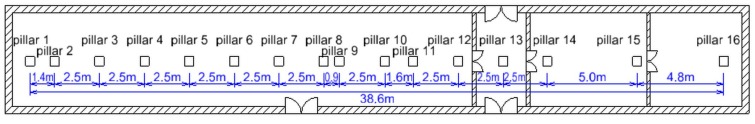
Schematic of the laboratory baseline.

The baseline is located in the geodetic basement laboratory in building C of the Faculty of Civil Engineering, Czech Technical University in Prague. The baseline is formed by 16 concrete pillars in one row (Figure 3). The height of the pillars is 0.9 m, the size of the square heads is 0.35 m by 0.35 m. The pillars are mutually spaced from 0.9 m to 5.0 m and the total length of the baseline is 38.6 m. A year-round temperature of approximately 20 °C is maintained in the laboratory.

**Figure 4:**
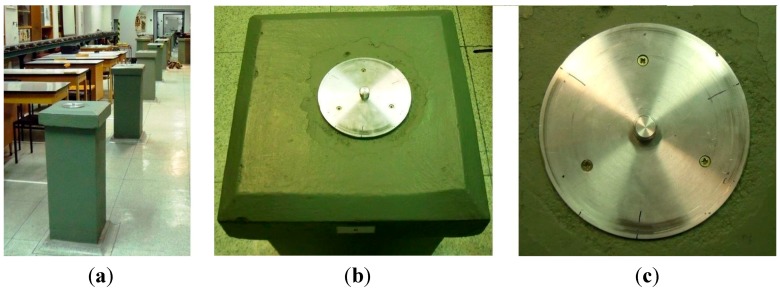
(**a**) The laboratory baseline; (**b**) the head of the pillar; (**c**) the centering plate.

In 2013, the heads of the pillars were equipped with centering plates (Figure 4) that are lined up with a maximum transverse deviation of 2 mm. Each centering plate is leveled in the horizontal plane (maximum slope of 0.7%). The heights of the centering plates are not the same due to the different heights of the pillars; the maximum elevation is 50 mm. Centering plates are machine-cut from hardened duralumin cylinders. The plate diameter is 140 mm and in the middle is the clamping screw. The plates are bolted to the head of the pillars at three points with 40 mm screws with dowels. A line is marked on each centering plate to ensure the appropriate tightening of the tribrach to the same position.

### 2.3. Choice and Testing of the Measurement Accessories

The character of the laboratory does not allow having tribrachs mounted permanently on all of the pillars; therefore, an option was chosen of selecting two tribrachs, one carrier and one prism that would be reserved for measurement of the base, and not used for other activities to ensure that they would be kept intact. The aim was to find tools that would suffer from minimum effects of eccentricity and that would guarantee the same position (position repeatability) for all measurements on the base.

**Figure 5:**
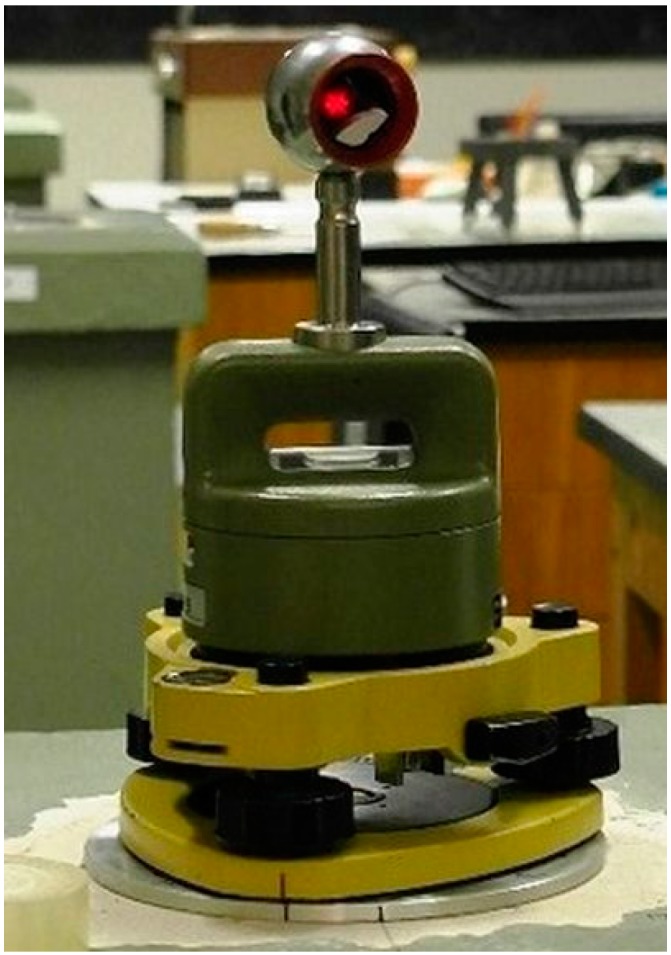
The test kit–tribrach Topcon, carrier Leica GZR3, Leica prism RRR 1.5″.

For the first tests at the base in 2013, five tribrachs, Topcon, Leica carrier GZR3 and 3 miniprisms Leica GMP101, were used. For testing, reference Leica Absolute Tracker AT401 and spherical prism Leica RRR 1.5″ were used (Figure 5).

All tests were based on multiple repetitions, and the averaged values and the standard deviation were determined from the Equation:

(3) $$s=\sqrt{\frac{1}{n-1}\cdot \sum_{i=1}^{n}{\left(\bar{x}-x_{i}\right)}^{2}}$$

where *n* is the sample size, *x_i_* is *i*-th measured value, and $\bar{x}$ is the arithmetic mean.

To verify the reliability and proper functioning of the tribrachs, tests of repeated centering of the tribrach with the carrier and repeated positioning of the carrier itself were designed. The Leica Tracker was positioned on a forced centering on pillar No. 1. Five tribrachs were successively tested on pillar No. 3 (at distance 3.9 m). To test repeatability, centration was always applied to the appropriate tribrach and carrier together. Screwing on of the centering plate and leveling by the carrier bubble was performed five times; tribrach screw positions and carrier orientation were kept the same in all cases. After each screwing, the distance measurement was made. The maximum difference between tribrachs was 0.7 mm. The sample standard deviation of centration repeatability for each tribrach was between 0.002 mm and 0.007 mm. To test the repeatability of the clamping of the carrier (a functional verification of the tribrach locks) the tribrach was always screwed to the centering plate and leveled by the carrier bubble. Then, the carrier was removed and clamped again five times with unchanged leveling and orientation of the carrier. After each clamping of the carrier, the distance was measured. Clamping repeatability standard deviation values were from 0.002 mm to 0.05 mm. Based on these tests, two tribrachs were selected which showed similar error of centration in the test and had a minimum standard deviation of carrier clamping to the tribrach. One of the leveling screws on each tribrach was glued to fix the tribrach height.

The eccentricity of the carrier was also tested. The carrier was first leveled in the tribrach. Then, the carrier was rotated around the vertical axis in nine positions (two times, clockwise and counter-clockwise). In each position the horizontal distance and the horizontal direction were measured. Based on this experiment, approximately circular eccentricity was detected, with a radius 0.03 mm.

Additive constants for reflecting prisms were determined by the comparison of distances measured on the spherical prism Leica RRR1.5″ and mini prisms Leica GMP101, five times each (independently). For all tested prisms, the additive constant was determined to be approximately −16.5 mm compared to the −16.9 mm specified by the manufacturer. The sample standard deviation of the additive constants was between 0.006 and 0.03 mm. Based on the test, one prism was selected and the determined additive constant was thereafter used.

In 2014, new test measurements of two new Leica tribrachs and one new carrier Leica GZR3 were made. New accessories were also tested using the previous procedure. The difference in the centers of the tribrachs was found to be 0.3 mm. The sample standard deviation of centration repeatability for each tribrach was between 0.006 and 0.01 mm. For a new carrier, circular eccentricity with a radius of 0.03 mm was detected. For the used mini prism Leica GMP101, a new additive constant due to new tribrachs and a carrier was determined, (difference 0.05 mm compared to the old one).

**Figure 6:**
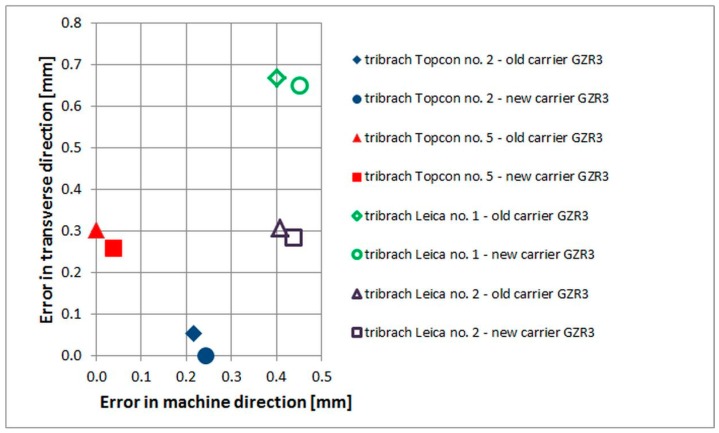
Comparison of the tribrachs and carriers centers on one forced centering.

The values found during the test measurements reflect the wear on the accessories and point to the need for increased control of used equipment. The results also show that even when using forced centering and precise tools, the eccentricities (Figure 6) influence the results, and an appropriate measurement procedure has to be used to reduce them.

### 2.4. Determination of the Absolute Lengths of the Laboratory Baseline

To test the distance meters at the baseline, it was necessary to determine the horizontal distance between the pillars with an accuracy of better than 0.05 mm. Given the results of previous testing of the accessories, it was necessary to introduce rules that suppress the effects of possible eccentricities.

Lines were marked on the tribrachs and centering plates to ensure the same clamping of the tribrachs. One of the screws on each tribrach had been glued to ensure that, after the leveling, the tribrach will always be at the same height. The rotation of the eyepiece optical plummet of the carrier was defined to be always toward pillar No. 1. One tribrach was dedicated only for the tested instrument at pillar No. 1. The second tribrach was dedicated for the other 15 pillars with the carrier Leica GZR3. The carrier was always placed on the tribrach in the same way. Spherical prism Leica RRR1.5″, which has always been in the same position (Leica sign up), was used for determining the dimensions of the baseline, and Prism Leica GMP101 was used for the practical test measurements. This prism has always been rotated toward pillar No. 1. The additive constant was determined for the set carrier Leica GZR3 and prism Leica GMP101.

In July 2013, the first determination of the absolute lengths of the baseline by the Leica Absolute Tracker AT401 and accessories described above was carried out. The measurement was carried out from the first, the last, and the middle pillars (No. 1, No. 16, and No. 10), and from one station out of the pillar, 1.5 m from the baseline axis near pillar No. 6. Measurements were taken from each station at every other point of the baseline one time in both faces of the telescope.

Local coordinates were calculated from the measured horizontal directions and horizontal distances by the least square adjustment [23]; there were 122 observations (61 directions, 61 distances), of which 81 were redundant. *A priori* standard deviations for the adjustment were selected; 0.3 mgon for directions and 0.025 mm for distances. The *a priori* standard unit deviation was selected to be 1.00 and the *a posteriori* was 0.77, which means that the measurement was more precise than was assumed. The horizontal distances between pillars were calculated from the coordinates, with standard deviations of 0.02 mm.

In January 2014, a control measurement was carried out, which confirmed the overall stability of the base. Additionally, new reference lengths were determined for the new tribrachs and the carrier. Determination of the new lengths was by a similar procedure to that of in July 2013 and by the same instrument, Leica AT 401, but only from the 3 stations on pillars No. 1, No. 10, and No. 16. There were 90 observations in total (45 directions, 45 distances), and 54 were redundant. Standard deviations of 0.15 mgon were used for the horizontal directions and 0.020 mm for the distances. The *a priori* standard unit deviation was 1.00, *a posteriori* 0.69, and the horizontal distances between pillars were calculated from the coordinates with standard deviations of 0.015 mm.

In July 2014, another check measurement was made, which was carried out in exactly the same manner as in January 2014. Similar results were obtained as in the previous measurement. Reference lengths were recalculated for the current accessories and they were still characterized by a standard deviation of 0.015 mm; thus, this baseline, characterized by its stability and accuracy, is suitable for subsequent accurate testing of the distance meters of the total stations.

### 2.5. Determination of the Size of the Absolute Errors of Selected Distance Meters and Their Stability over Time

To determine the absolute size of the error (ε_distance_) and to calculate the correction it is important to verify the repeatability of the measurement and its stability over time. The first tests were conducted to verify these assumptions. The tests are based on measuring the distance from the first pillar to all other pillars (*d_i_*, 15 lengths) and comparing the measured distances with nominal values (*D_i_*).

(4) $${\text{ε}}_{\text{i,distance}}=D_{i,reference}-d_{i,measured}$$

Time intervals for verification of constancy were selected in from weeks to months, and between each test the instruments were used normally.

Within the test measurement, the above-mentioned fundamental rules were respected. The total station was inserted to the tribrach in the same rotation, and leveling was performed with electronic levels. The additive constant was set to 0 mm in the instrument and the physical corrections were also set. The temperature and the pressure of the air were measured in the middle of the measured distance. Zenith angles and slope distances (with a resolution at least 0.1 mm) were registered during measurements. Every instrument was tempered to the immediate environment temperature for 30 min. Warm up of the instrument by performing 50 distance measurements was carried out before the test. The instruments used are factory-build (without modification) and there is not solved compensation of the temperature changes of individual components, as described, e.g., in [24,25,26]. Measuring of individual distances was executed in *n* repetitions in face I (without retargeting) and then in *n* repetitions in face II (without retargeting). The horizontal distances were calculated from the measured values, which were averaged, corrected with the additive constant and compared with the reference length. Sample standard deviations of the measurements were also calculated, as were the differences between the values measured in face I and face II.

#### Determination the Number of Repetition of the Measurements

The number *n* of repeated distance measurements in one position of the telescope is determined on the basis of considerations about the value of the standard deviation of the standard deviation of the measured distance σ_d_ [27]. It is given by Equation (5):

(5) $${\text{σ}}_{d}=\frac{\text{σ}}{\sqrt{2\cdot \left(n-1\right)}}$$

where σ is the standard deviation of the measured distance. On the basis of the chosen conditions, that standard deviation σ_d_ can be maximally 10% of σ; then the size *n* of the range of random sampling is given by:

(6) $${\text{σ}}_{d}=\frac{\text{σ}}{\sqrt{2\cdot \left(n-1\right)}}=0.1\cdot \text{σ}\Rightarrow n=51$$

Due to this condition, 102 measured values for each distance meter and each distance are acquired. The sample standard deviation *s_d_* of the averaged distance is given by Equation (7):

(7) $$s_{d}=\sqrt{\frac{1}{2\cdot n-1}\cdot \sum_{i=1}^{2n}{\left(\bar{d}-d_{i}\right)}^{2}}$$

where $\bar{d}$ is the average distance and *d_i_* is *i*-th measured distance.

### 2.6. Determination of the Relative Errors of Selected Distance Meters in Detail Using the Baseline with an Interferometer

To create a correction function, it is necessary to know the detailed course of absolute error of the measurement over the whole range of application of the correction function. It cannot be determined using the baseline with pillars, and it is necessary to combine results from absolute and relative baseline.

**Figure 7:**
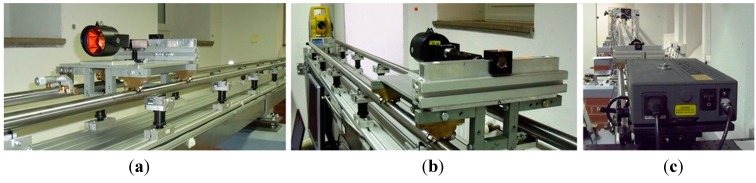
Baseline with interferometer (TU Dresden): (**a**) the interferometer’s carriage with the prism; (**b**) the interferometer’s carriage with the prism and the total station; (**c**) the interferometer Renishaw ML10.

The baseline with an interferometer in the geodetic laboratory at TU Dresden was used for detailed relative testing. The laboratory base consists of a rail on which a carriage with prisms moves (Figure 7). The total station is placed at one end and at the other end is the interferometer, Renishaw ML10 (the standard deviation of the distance determination is 0.5 ppm). The entire length of the baseline is 25 m. Six pressure and temperature sensors for the implementation of physical corrections to the interferometer were equally spaced on the entire baseline length.

During the tests the physical corrections were entered into the total stations. The temperature and the pressure of the air were consistent along the baseline. During the tests, the distances were measured only in the first face in 16 repetitions. The number of repetitions was chosen with respect to the total measurement time and according to Equation (6), and the standard deviation of the sample standard deviation σ_d_ is maximally 20% of σ. It was measured at one face only because relative errors only are being determined. The relative errors were determined on the basis of the measured distance differences:

(8) $$\Delta_{i,\text{distance}}=\left(D_{EDM,origin}-D_{EDM,i}\right)-\left(D_{INTERFEROMETER,i}-D_{INTERFEROMETER,origin}\right)$$

where, *D_EDM,origin_* is the distance measured by the total station at the initial point, *D_INTERFEROMETER,origin_* is set to the initial point of the interferometer, and *D_EDM,i_*, *D_INTERFEROMETER,i_* are distances measured by the total station and by the interferometer, respectively, to the actual measured point.

A breakpoint was set at the beginning of the track near the interferometer, on which the initial point of measurement was set. This initial point was periodically checked during the tests. For each instrument, basic testing was performed; sections of 10 mm, 100 mm, and 2 m were measured in steps of 1 mm, 10 mm and 100 mm. On the basis of this test, the step for the whole baseline measurement was determined. The size of the variance of the errors and any possible trend, which should be identified and well-measured on whole baseline, was determined from the short-step measurements. The sizes of the variances corresponded to the standard deviations determined on the absolute baseline. After selecting the appropriate step, the interval from 2.2 to 24.2 m was measured (measured from the total station). The resulting data were smoothed using a moving average of the third grade.

### 2.7. Determination of the Correction Function from a Combination of Absolute and Relative Errors

For the complete determination of corrections, the absolute and relative errors of the measured distances must be merged. Data corresponding to data measured on the pillar baseline (the same absolute distance) were interpolated from the relative data. The mean difference was also calculated, which converts the relative data to absolute. Since all error values are affected by uncertainty, a robust average was used to calculate the shift. It was calculated iteratively and weights were calculated according to the L1 norm.

(9) $$w_{i}=\frac{1}{\left|{\text{ε}}_{i,\text{distance}}-\Delta_{i,\text{distance}}\right|}$$

(10) $$shift=\frac{\sum_{i=1}^{m}\left({\text{ε}}_{i,\text{distance}}-\Delta_{i,\text{distance}}\right)\cdot w_{i}}{\sum_{i=1}^{m}w_{i}}$$

where *ε_i,distance_* is an absolute error (pillar baseline measurement), *Δ_i,distance_* is a relative error (interferometric baseline measurement), both at the same distance, *m* is a number of differences and *w_i_* are corresponding weights.

Relative errors identified at the interferometric baseline were shifted and converted to absolute errors. From these absolute errors corrections of the measured distances can be calculated. The corrections are interpolated for the distance corresponding to the measured distances. It is used a linear interpolation of systematic errors between the distances from the baseline with interferometer.

The Fourier transform can also be applied on the absolute deviation, and the main parameters of harmonic functions (amplitude, wavelength, phase shift) determined. A correction function was also compiled from these parameters (in detail described in Section 3.5).

### 2.8. Experimental Verification of the Suitability and Correctness of the Distance Corrections

On the basis of the above experiments and their results, sets of deviations from which corrections for any measured distance that will fall within the specified interval of known absolute errors can be calculated were obtained. This procedure can be applied only to the fully tested distance meters (both absolute and interferometric baseline). To confirm the correctness of the procedure, an independent test was designed.

The experiment verifying the accuracy of the absolute errors’ identification procedure and the implementation of corrections was performed in the laboratory with the pillar baseline, but on different points. The test consisted of comparing distances (calculated from coordinates determined by the Leica Absolute Tracker AT401) with distances measured using total stations and with the corrected distances. The Leica Absolute Tracker AT401 was set up on pillar No. 1, with total stations successively on pillar No. 3. A local coordinate system was created for both the total station and the tracker by simultaneously measuring four reference points. From this measurement, the mutual position of both instruments was determined with high precision (less than 0.05 mm). The measured testing points were realized using the spherical prism Leica RRR 1.5″, mounted in a magnetic nest placed on the metal rail. Points were measured simultaneously by the tracker and the total station. From the calculated coordinates (in the local coordinate system described above) of points measured by the tracker, distances equivalent to the total station measured ones were calculated. Then, differences between distances determined by the total station (with and without applied corrections) and by the tracker were calculated. Testing points were placed at three intervals: 5–6 m, 12–13 m, and 19–20 m, all filled with points equally spaced in 50 mm steps. The distances were measured with 16 repetitions by the total station and in both faces of telescope. A measurement with the tracker was one repetition.

## 3. Results and Discussion

### 3.1. Laboratory Pillar Baseline

To test the distance meters used in precision industrial engineering surveying, the laboratory pillar baseline with forced centerings described above was built. Specially selected accessories that showed minimal negative influence of the eccentricities were used for the test. The whole baseline was repeatedly measured by the Leica Tracker AT401 and the coordinates of points on the pillars were determined by the least squares method. The resulting horizontal lengths were calculated from the coordinates; the standard deviation was less than 0.02 mm (Table 1). A stable high-precision laboratory pillar baseline for testing purposes was created. Lengths were determined at 24.5 °C.

**Table 1:** Length of laboratory pillar baseline (07/2014).

Length between Points	Length (m)	Standard Deviation (mm)
1–2	1.364207	0.012
1–3	3.861435	0.012
1–4	6.377912	0.012
1–5	8.865026	0.012
1–6	11.375357	0.012
1–7	13.865353	0.012
1–8	16.354823	0.012
1–9	17.224086	0.012
1–10	19.761757	0.015
1–11	21.329565	0.012
1–12	23.852714	0.012
1–13	26.359595	0.012
1–14	28.804906	0.012
1–15	33.805417	0.012
1–16	38.629900	0.015

### 3.2. Determination of the Sizes of the Systematic Errors of EDM Measurement and Their Stability over Time

The aim of the experiments was to test pulse and phase distance meters with standard deviations of the measured distance between 1 and 3 mm (for short distances). All instruments used were from the property of the Faculty of Civil Engineering, Czech Technical University in Prague (Table 2).

Below are the test results for the above-mentioned instruments and all epochs of testing. There are graphs representing the size of the absolute errors from the nominal length. Furthermore, there are also tables relating to the last epoch of the measurement, showing the difference between face I and II, the scattering of measured distances and the sample standard deviation of a single measurement (from a file with 102 values). For information about the size of random errors in the context of repeated measurements on a single pillar in one epoch, there are graphs representing the differences of the measured distances from the average distance. All tests were carried out under practically the same temperature 24.5 °C (±1 °C). The instruments are presented according to the number of the realized testing epochs.

**Table 2:** Overview of tested instruments.

Instrument	Distance Meter Type	Unit Length	Standard Deviation σ_D_
Leica TC1202	Phase	1.5 m	2 mm + 2 ppm
Leica TS06	Phase	1.5 m	1.5 mm + 2 ppm
Leica TC1800	Phase	3.0 m	1 mm + 2 ppm
Leica TC2003	Phase	3.0 m	1 mm + 1 ppm
Topcon GPT-7501	Pulse	-	2 mm + 2 ppm
Trimble S6 HP	Phase	0.37 m	1 mm + 1 ppm
Trimble S8	Phase	0.37 m	0.8 mm + 1 ppm
Trimble M3	Pulse	-	3 mm + 2 ppm

#### 3.2.1. Trimble S6 HP

**Figure 8:**
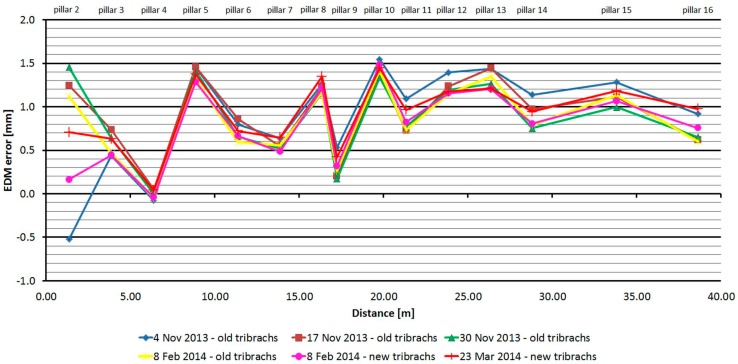
Trimble S6 HP–Absolute errors in six epochs.

The Trimble S6 HP was tested six times over five months. As is shown at Figure 8, the average distances differ between epochs, maximally, by 0.4 mm and thus good repeatability of measured distance and its stability over time was achieved. Differences between the epochs can be caused by measurement errors, including EDM errors, leveling of accessories, centration errors, and other operator errors. The distance of pillar No. 2 shows the maximum difference of up to 2 mm between the epochs; this difference is probably due to the very short length (1.36 m). The resulting difference from the nominal length is affected by the cyclic error of the phase EDM, and the residual error of the additive prism constant also contributes a certain share. The standard deviation of a single measurement is 0.3 mm and is not dependent on the measured distance (Table 3).

**Figure 9:**
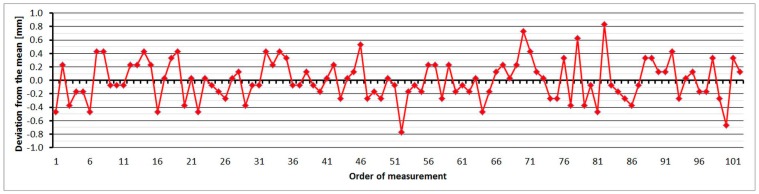
Trimble S6 HP–Differences from average distance–pillar 7 (13.9 m).

The average distance difference between face I and face II is not systematic; the average value is 0.04 mm, which is below the resolution of the EDM. Comparing deviations from the mean (Figure 9) shows that the EDM has a large amount of random errors, with a difference between maximum and minimum of up to 1.6 mm. The singly-measured distance is therefore unreliable and, for correct results, it is necessary to choose repeated measurement of distances to suppress random errors.

**Table 3:** Trimble S6 HP (23 February 2014).

Length from Pillar 1 to	Nominal Length (m)	EDM Error (mm)	Sample std. dev. (mm)	Difference between Faces (mm)	Maximum–Minimum Difference (mm)
**Pillar 2**	1.364	0.71	0.52	0.53	2.50
**Pillar 3**	3.861	0.63	0.29	−0.01	1.60
**Pillar 4**	6.378	0.04	0.33	0.23	1.60
**Pillar 5**	8.865	1.38	0.37	0.26	1.50
**Pillar 6**	11.375	0.72	0.29	−0.07	1.30
**Pillar 7**	13.865	0.65	0.30	0.00	1.60
**Pillar 8**	16.354	1.35	0.28	−0.13	1.40
**Pillar 9**	17.224	0.42	0.32	−0.24	1.30
**Pillar 10**	19.761	1.45	0.25	0.01	1.50
**Pillar 11**	21.329	0.96	0.31	−0.01	1.50
**Pillar 12**	23.852	1.18	0.26	−0.03	1.20
**Pillar 13**	26.359	1.21	0.29	−0.06	1.60
**Pillar 14**	28.805	0.94	0.30	0.16	1.40
**Pillar 15**	33.805	1.18	0.26	0.02	1.20
**Pillar 16**	38.630	0.97	0.31	−0.01	1.40

#### 3.2.2. Leica TC1202

**Figure 10:**
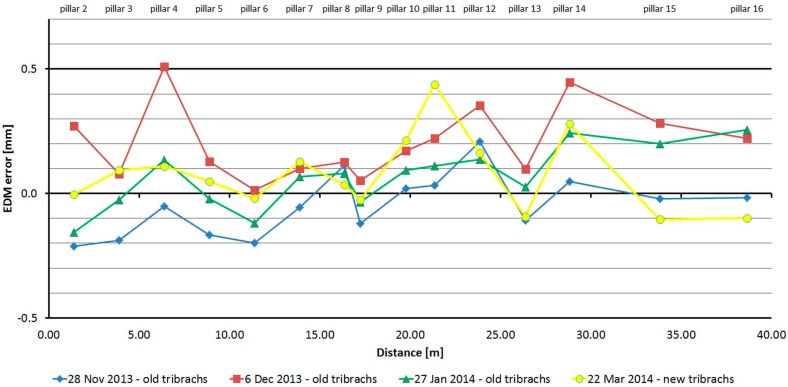
Leica TC1202–Absolute errors in four epochs.

The Leica TC1202 was tested four times over five months. As is shown at Figure 10, the average distances differ between epochs, maximally, by 0.4 mm (except pillar No. 2 and No. 4, where the difference is 0.5 mm; this was probably caused by operator error in leveling), and thus, good repeatability of measured distance and its stability over time was achieved. The resulting errors oscillate around 0 mm, and their size is mainly affected by the cyclic error of the phase EDM. The standard deviation of a single measurement is 0.1 mm and is not dependent on the measured distance (Table 4).

**Figure 11:**
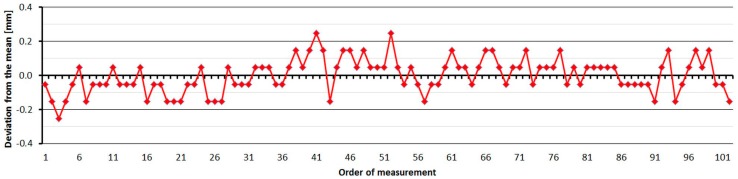
Leica TC1202–Differences from average distance–pillar 7 (13.9 m).

The average distance difference between face I and face II is 0.05 mm, which is below the resolution of the EDM. Comparing deviations from the mean (Figure 11) shows that the EDM has random errors with a difference between maximum and minimum of up to 0.5 mm. Singly-measured distances are therefore unreliable and, for correct results, it is necessary to choose repeated measurement of distances to suppress random errors.

**Table 4:** Leica TC1202 (22 March 2014).

Length from Pillar 1 to	Nominal Length (m)	EDM Error (mm)	Sample std. dev. (mm)	Difference between Faces (mm)	Maximum–Minimum Difference (mm)
**Pillar 2**	1.364	0.00	0.16	0.25	0.70
**Pillar 3**	3.861	0.09	0.10	0.06	0.50
**Pillar 4**	6.378	0.11	0.09	0.08	0.50
**Pillar 5**	8.865	0.05	0.11	0.12	0.50
**Pillar 6**	11.375	−0.02	0.10	0.04	0.60
**Pillar 7**	13.865	0.13	0.10	0.04	0.50
**Pillar 8**	16.354	0.04	0.10	0.06	0.60
**Pillar 9**	17.224	−0.03	0.09	0.03	0.30
**Pillar 10**	19.761	0.21	0.10	0.08	0.50
**Pillar 11**	21.329	0.44	0.10	0.02	0.50
**Pillar 12**	23.852	0.16	0.09	0.02	0.50
**Pillar 13**	26.359	−0.09	0.11	0.04	0.50
**Pillar 14**	28.805	0.28	0.10	0.06	0.40
**Pillar 15**	33.805	−0.10	0.11	0.02	0.50
**Pillar 16**	38.630	−0.10	0.11	0.04	0.50

#### 3.2.3. Trimble M3

**Figure 12:**
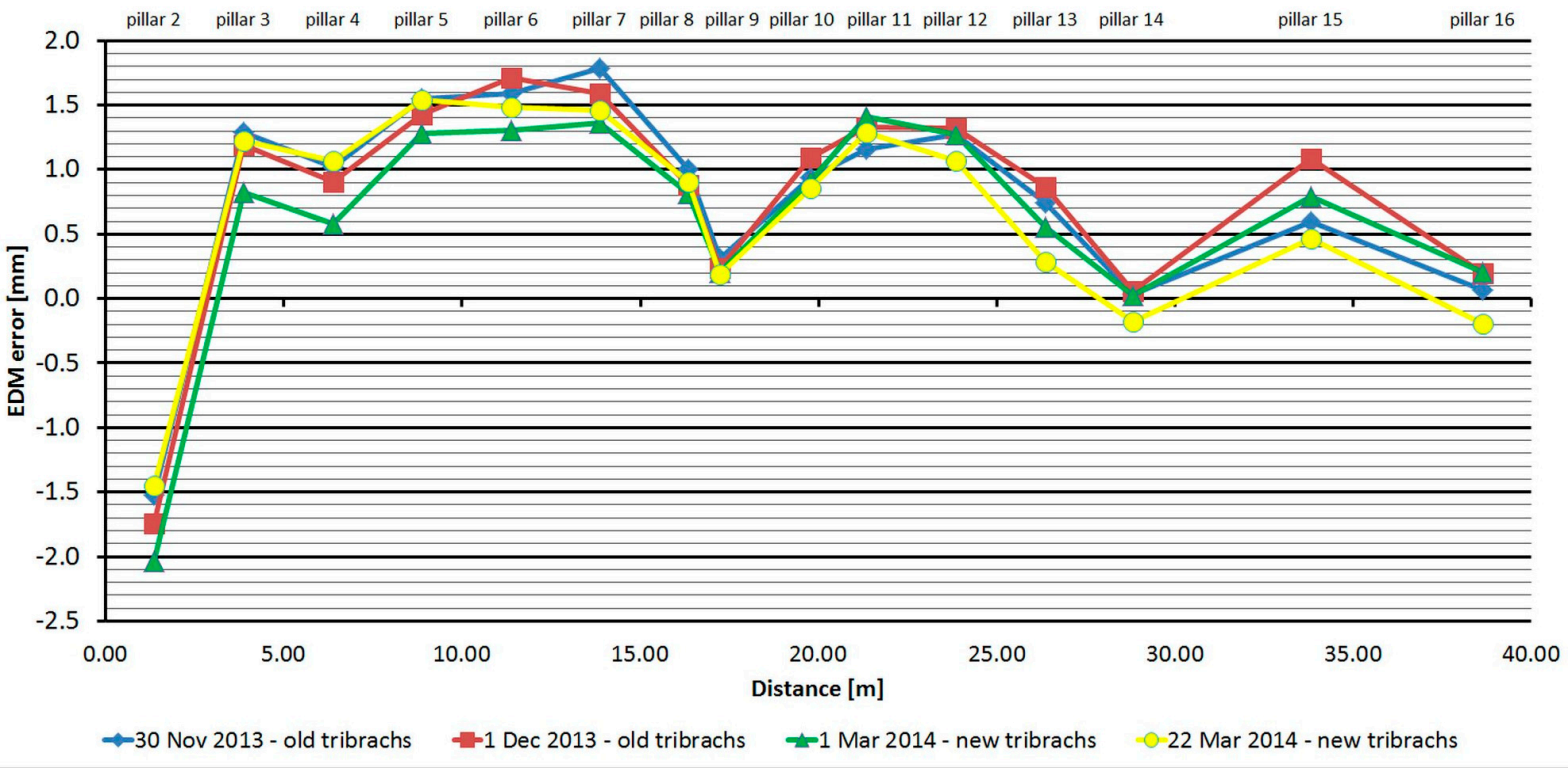
Trimble M3–Absolute errors in four epochs.

The Trimble M3 was tested four times over five months. As is shown at Figure 12, average distances differ between epochs, maximally, by 0.6 mm, and thus good repeatability of measured distance and its stability over time was achieved. The resulting difference from the nominal length is affected by the change of error of the additive constant. The standard deviation of a single measurement is 0.4 mm and is not dependent on the measured distance (Table 5).

**Table 5:** Trimble M3 (22 March 2014).

Length from Pillar 1 to	Nominal Length (m)	EDM Error (mm)	Sample std. dev. (mm)	Difference between Faces (mm)	Maximum–Minimum Difference (mm)
**Pillar 2**	1.364	−1.45	0.36	−0.10	1.88
**Pillar 3**	3.861	1.23	0.43	−0.35	2.00
**Pillar 4**	6.378	1.07	0.42	0.30	1.80
**Pillar 5**	8.865	1.54	0.45	0.15	2.60
**Pillar 6**	11.375	1.49	0.46	0.20	2.20
**Pillar 7**	13.865	1.46	0.53	−0.01	2.30
**Pillar 8**	16.354	0.90	0.45	0.13	2.00
**Pillar 9**	17.224	0.19	0.45	−0.14	1.80
**Pillar 10**	19.761	0.86	0.42	−0.19	2.00
**Pillar 11**	21.329	1.29	0.42	0.23	2.50
**Pillar 12**	23.852	1.07	0.44	0.08	2.30
**Pillar 13**	26.359	0.29	0.51	−0.63	2.10
**Pillar 14**	28.805	−0.18	0.51	−0.27	2.30
**Pillar 15**	33.805	0.46	0.49	0.42	2.80
**Pillar 16**	38.630	−0.19	0.46	0.15	2.20

**Figure 13:**
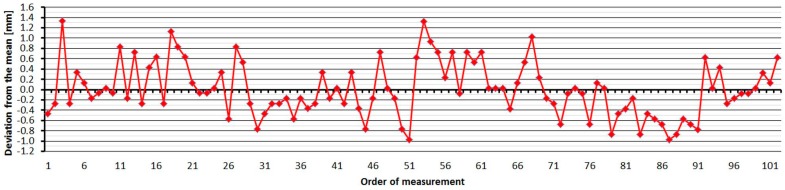
Trimble M3–Differences from average distance–pillar 7 (13.9 m).

The average distance difference between face I and face II is 0.1 mm, which is below the resolution of the EDM. Comparing deviations from the mean (Figure 13) shows that the EDM has random errors with a difference between maximum and minimum of up to 2.5 mm. Singly-measured distances are therefore unreliable and, for correct results, it is necessary to choose repeated measurement of distances to suppress random errors.

#### 3.2.4. Topcon GPT-7501

**Figure 14:**
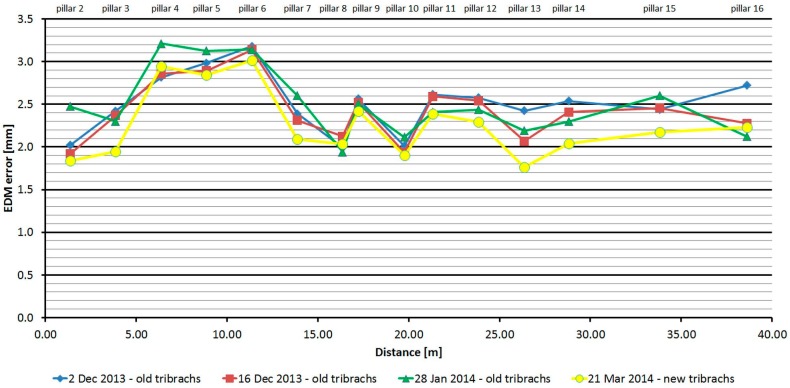
Topcon GPT-7501–Absolute errors in four epochs.

The Topcon GPT-7501 was tested four times over four months. As is shown at Figure 14, the average distances differ between epochs, maximally, by 0.6 mm, and thus good repeatability of measured distance and its stability over time was achieved. The resulting difference from the nominal length is affected by the change of error of the additive constant and its development (in connection with the time element) for the first 15 meters. The standard deviation of a single measurement is 0.2 mm and is not dependent on the measured distance (Table 6).

**Figure 15:**
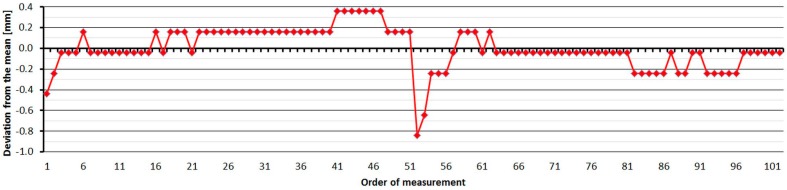
Topcon GPT-7501–Differences from average distance–pillar 7 (13.9 m).

The average distance difference between face I and face II is −0.2 mm (which corresponds to the resolution of the EDM), and thus, for a correct determination of distance, it should be measured in both positions of the telescope. Comparing deviations from the mean (Figure 15) shows that the EDM has random errors with a difference between maximum and minimum of up to 1.2 mm. Singly-measured distances are therefore unreliable and, for correct results, it is necessary to choose repeated measurement of distances to suppress random errors. The Topcon GPT-7501 EDM also has a change in distance after the beginning of the measurement; the first measurement is longer than for subsequent repetitions, and for this reason it is necessary to increase the number of repetitions of the measurement and exclude the first one.

**Table 6:** Topcon GPT-7501 (28 January 2014).

Length from Pillar 1 to	Nominal Length (m)	EDM Error (mm)	Sample std. dev. (mm)	Difference between Faces (mm)	Maximum–Minimum Difference (mm)
**Pillar 2**	1.364	2.47	0.33	−0.51	1.00
**Pillar 3**	3.861	2.30	0.39	−0.69	1.00
**Pillar 4**	6.378	3.21	0.22	−0.23	0.80
**Pillar 5**	8.865	3.13	0.22	−0.30	1.00
**Pillar 6**	11.375	3.14	0.17	−0.23	0.60
**Pillar 7**	13.865	2.60	0.20	−0.22	1.00
**Pillar 8**	16.354	1.94	0.15	0.11	0.80
**Pillar 9**	17.224	2.51	0.15	−0.09	1.00
**Pillar 10**	19.761	2.12	0.24	−0.40	0.60
**Pillar 11**	21.329	2.41	0.18	−0.12	0.80
**Pillar 12**	23.853	2.44	0.17	−0.26	0.40
**Pillar 13**	26.359	2.19	0.19	−0.19	1.00
**Pillar 14**	28.805	2.30	0.20	−0.26	0.60
**Pillar 15**	33.805	2.60	0.19	−0.23	0.60
**Pillar 16**	38.630	2.12	0.28	0.37	0.80

#### 3.2.5. Leica TS06

**Figure 16:**
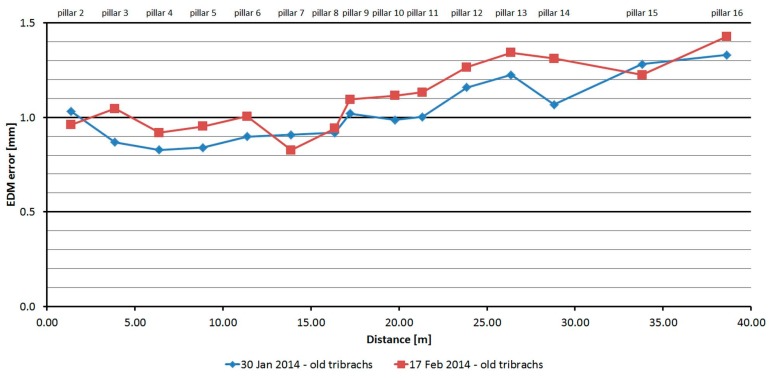
Leica TS06–Absolute errors in two epochs.

The Leica TS06 was tested two times over two months. As is shown at Figure 16, the average distances differ between epochs, maximally, by 0.2 mm, and thus good repeatability of measured distance and its stability over time was achieved. The resulting errors up to the distance of 15 m are constant and then start to grow. Their size is affected by the error of the additive constant and less by the cyclic error. The standard deviation of a single measurement is 0.1 mm and is not dependent on the measured distance (Table 7).

**Figure 17:**
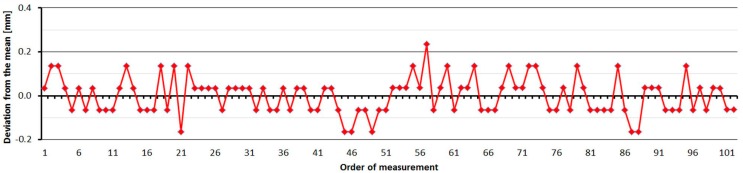
Leica TS06–Differences from average distance–pillar 7 (13.9 m).

The average distance difference between face I and face II is 0.1 mm (it corresponds to the resolution of the EDM) and thus, for a correct determination of distance, it should be measured in both positions of the telescope. Comparing deviations from the mean (Figure 17) shows that the EDM has random errors with a difference between maximum and minimum of up to 0.5 mm. Singly-measured distances are therefore unreliable and, for correct results, it is necessary to choose repeated measurement of distances to suppress random errors.

**Table 7:** Leica TS06 (27 February 2014).

Length from Pillar 1 to	Nominal Length (m)	EDM Error (mm)	Sample std. dev. (mm)	Difference between Faces (mm)	Maximum–Minimum Difference (mm)
**Pillar 2**	1.364	0.96	0.13	0.19	0.51
**Pillar 3**	3.861	1.05	0.10	0.14	0.50
**Pillar 4**	6.378	0.92	0.09	0.04	0.40
**Pillar 5**	8.865	0.95	0.09	0.07	0.40
**Pillar 6**	11.375	1.00	0.10	0.08	0.50
**Pillar 7**	13.865	0.83	0.08	0.02	0.40
**Pillar 8**	16.354	0.94	0.10	−0.05	0.40
**Pillar 9**	17.224	1.09	0.09	0.02	0.20
**Pillar 10**	19.761	1.11	0.10	0.07	0.50
**Pillar 11**	21.329	1.13	0.12	0.14	0.50
**Pillar 12**	23.853	1.27	0.11	0.10	0.40
**Pillar 13**	26.359	1.34	0.11	0.14	0.50
**Pillar 14**	28.805	1.31	0.16	0.25	0.60
**Pillar 15**	33.805	1.23	0.09	0.05	0.50
**Pillar 16**	38.630	1.43	0.12	0.15	0.50

#### 3.2.6. Trimble S8

**Figure 18:**
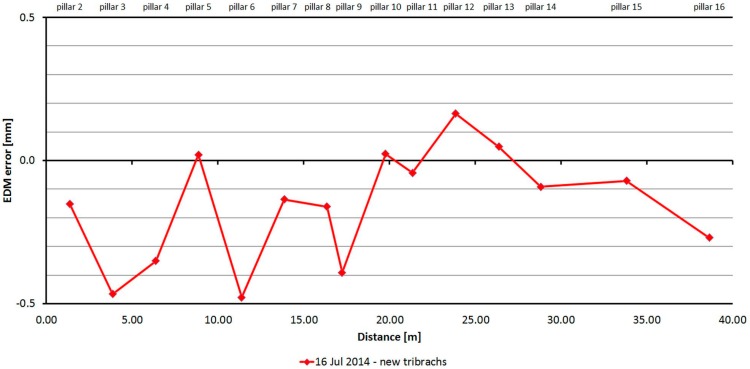
Trimble S8–Absolute errors in one epoch.

The Trimble S8 was tested just one time. As is shown at Figure 18, distances differ from nominal ones from −0.5 to 0.3 mm and are caused mainly by the cyclic error of the EDM. The standard deviation of a single measurement is 0.3 mm and is not dependent on the measured distance (Table 8).

**Figure 19:**
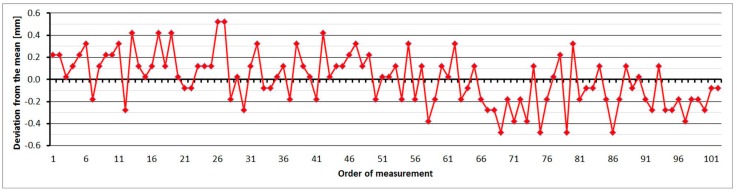
Trimble S8–Differences from average distance–pillar 7 (13.9 m).

The average distance difference between face I and face II is −0.1 mm (which corresponds to the resolution of the EDM, which is 0.1 mm), and thus, for a correct determination of distance, it should be measured in both positions of the telescope. Comparing deviations from the mean, Figure 19 shows that the EDM has random errors with a difference between maximum and minimum of up to 1.5 mm. Singly-measured distances are therefore unreliable and, for correct results, it is necessary to choose repeated measurement of distances to suppress random errors.

**Table 8:** Trimble S8 (16 July 2014).

Length from Pillar 1 to	Nominal Length (m)	EDM Error (mm)	Sample std. dev. (mm)	Difference between Faces (mm)	Maximum–Minimum Difference (mm)
**Pillar 2**	1.364	−0.15	0.31	0.19	1.50
**Pillar 3**	3.861	−0.47	0.21	−0.05	1.00
**Pillar 4**	6.377	−0.35	0.22	−0.06	1.00
**Pillar 5**	8.865	0.02	0.24	−0.05	1.20
**Pillar 6**	11.375	−0.48	0.22	−0.10	1.20
**Pillar 7**	13.865	−0.14	0.24	−0.23	1.00
**Pillar 8**	16.354	−0.16	0.26	−0.23	1.30
**Pillar 9**	17.224	−0.39	0.25	−0.17	1.20
**Pillar 10**	19.761	0.02	0.26	−0.03	1.40
**Pillar 11**	21.329	−0.04	0.23	−0.10	1.10
**Pillar 12**	23.852	0.16	0.23	−0.07	1.20
**Pillar 13**	26.359	0.05	0.27	−0.34	1.20
**Pillar 14**	28.804	−0.09	0.24	−0.03	1.20
**Pillar 15**	33.805	−0.07	0.28	−0.28	1.60
**Pillar 16**	38.629	−0.27	0.31	−0.31	1.50

#### 3.2.7. Leica TC1800

**Figure 20:**
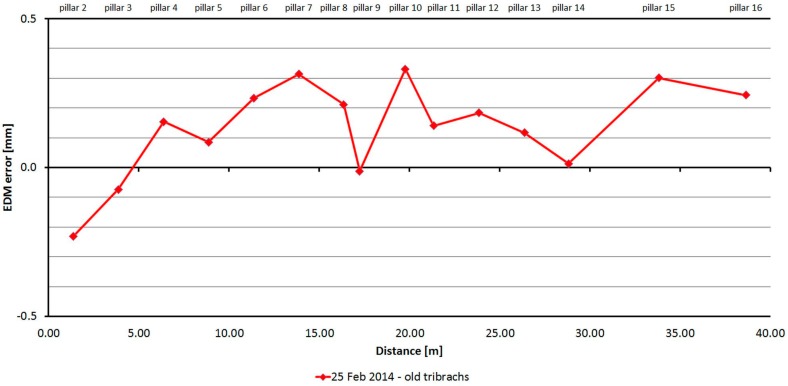
Leica TC1800–Absolute errors in one epoch.

The Leica TC1800 was tested just one time. As is shown at Figure 20, distances differ from nominal ones from −0.2 to 0.3 mm and are caused mainly by the cyclic error of the EDM. The standard deviation of a single measurement is 0.2 mm and is not dependent on the measured distance (Table 9).

**Table 9:** Leica TC1800 (25 February 2014).

Length from Pillar 1 to	Nominal Length (m)	EDM Error (mm)	Sample std. dev. (mm)	Difference between Faces (mm)	Maximum–Minimum Difference (mm)
**Pillar 2**	1.364	−0.23	0.41	−0.18	1.00
**Pillar 3**	3.861	−0.07	0.47	0.53	1.00
**Pillar 4**	6.378	0.15	0.00	0.00	0.00
**Pillar 5**	8.865	0.08	0.20	0.08	1.01
**Pillar 6**	11.375	0.23	0.43	0.46	1.00
**Pillar 7**	13.865	0.31	0.22	0.10	1.00
**Pillar 8**	16.354	0.21	0.22	0.10	1.01
**Pillar 9**	17.224	−0.01	0.00	0.01	0.00
**Pillar 10**	19.761	0.33	0.14	0.05	1.01
**Pillar 11**	21.329	0.14	0.00	0.01	0.01
**Pillar 12**	23.853	0.18	0.50	0.48	1.01
**Pillar 13**	26.359	0.12	0.14	0.04	1.00
**Pillar 14**	28.805	0.01	0.36	0.18	1.01
**Pillar 15**	33.805	0.30	0.50	0.51	1.00
**Pillar 16**	38.630	0.24	0.50	0.46	1.01

**Figure 21:**
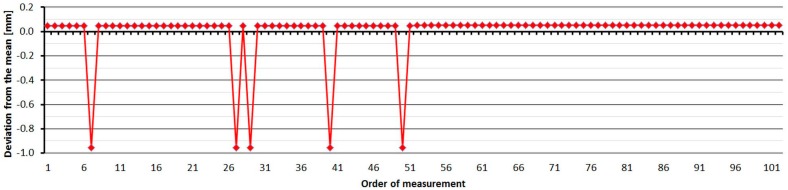
Leica TC1800–Differences from average distance–pillar 7 (13.9 m).

The average distance difference between face I and face II is 0.2 mm, and thus, for a correct determination of distance, it should be measured in both positions of the telescope. Comparing deviations from the mean, Figure 21 shows that although the EDM’s resolution is 0.1 mm, the measured value is either unchanged or changes by 1 mm. Singly-measured distances are therefore unreliable and, for correct results, it is necessary to choose repeated measurement of distances to suppress random errors.

#### 3.2.8. Leica TC2003

**Figure 22:**
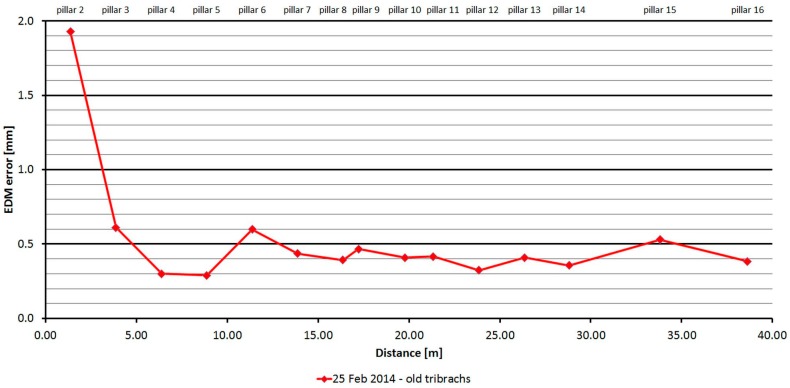
Leica TC2003–Absolute errors in one epoch.

The Leica TC2003 was tested just one time. As is shown at Figure 22, the distances differ from the nominal ones constantly by 0.4 mm. The main error is caused by the error of the additive constant. The standard deviation of a single measurement is 0.06 mm and is not dependent on the measured distance (Table 10).

**Figure 23:**
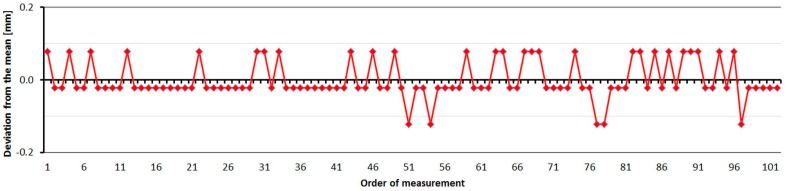
Leica TC2003–Differences from average distance–pillar 7 (13.9 m).

The test results confirmed the suitability of the instrument for determining of the calibration bases. For this purpose it was (and still is) used in many countries [10]. The average distance difference between face I and face II is smaller than 0.1 mm. Comparing deviations from the mean, Figure 23 shows that the EDM has random errors with a difference between maximum and minimum of up to 0.2 mm, and the measured value is either unchanged or changes by 0.1 mm.

**Table 10:** Leica TC2003 (25 February 2014).

Length from Pillar 1 to	Nominal Length (m)	EDM Error (mm)	Sample std. dev. (mm)	Difference between Faces (mm)	Maximum–Minimum Difference (mm)
**Pillar 2**	1.364	1.93	0.06	−0.02	0.30
**Pillar 3**	3.861	0.61	0.05	−0.03	0.20
**Pillar 4**	6.378	0.30	0.07	0.02	0.30
**Pillar 5**	8.865	0.29	0.06	−0.01	0.20
**Pillar 6**	11.375	0.60	0.06	0.01	0.30
**Pillar 7**	13.865	0.44	0.05	0.00	0.20
**Pillar 8**	16.354	0.39	0.06	0.01	0.20
**Pillar 9**	17.224	0.47	0.05	−0.01	0.20
**Pillar 10**	19.761	0.41	0.05	0.00	0.20
**Pillar 11**	21.329	0.42	0.07	0.00	0.50
**Pillar 12**	23.853	0.32	0.06	−0.04	0.30
**Pillar 13**	26.359	0.41	0.05	0.02	0.20
**Pillar 14**	28.805	0.36	0.06	−0.02	0.30
**Pillar 15**	33.805	0.53	0.06	−0.03	0.30
**Pillar 16**	38.630	0.38	0.05	0.01	0.30

#### 3.2.9. Assessment of Repeated Tests on the Laboratory EDM Pillar Baseline

The results of testing eight different instruments with different distance meters on the laboratory pillar baseline reveal some important facts. The repeated distance measurements of the same instrument under the same conditions and at the exact same points result almost in the same distances with only minor deviations (in tenths of a millimeter), which are caused by unavoidable residual systematic errors. These systematic errors, in this case, may be due not only to the instrument itself but also to other accessories used. The sizes of the absolute errors (the difference between the reference and the measured distance) are not the same for all distances and have a very variable size. In the case of the phase distance meters, cyclic error is the main source of errors and there is also influence from the additive constant error. In the case of the pulse distance meters, the additive constant error is the main source of errors, but it is variable for the first 15 m and then, with increasing distance, becomes approximately constant.

Another important finding is that, for all distance meters, repeated measurements of one distance causes random errors and deviations to occur at approximately 2.5 mm. The sizes of the deviations from the mean distance for each EDM in none of the cases exceeded the accuracy claimed by the manufacturer. This fact implies the need to carry out multiple distance measurements (preferably in both positions of the telescope) to suppress the influence of random errors and to gain more precise distance measurements. Notably, if instruments have been tested multiple times, the absolute errors of the average measured distances are always stable over time.

These findings support the idea of implementing systematic error correction of EDM-measured distances based on the measured distance and complex functions, rather than only by the additive constant and a constant dependent on the distance (as for long distances [4,8,15]). To determine the correction function, it is necessary have detailed knowledge of the absolute size of the errors. This can be advantageously determined by combining the absolute and relative errors from the absolute pillar baseline and the baseline with an interferometer.

### 3.3. Comparison of the Absolute Errors of the Same Type of EDM

The first test proved the stability of EDM errors over time and, therefore, a test to determine whether the same types of EDM have the same errors was designed. Four instrument series were tested. Six Topcon GPT-7501 instruments, six Trimble M3 instruments, two Leica TC1202 instruments, and two Leica TS06 instruments were tested. Instruments of the same type, belonging to the same production series and based on the production numbers, were made at the same time. The instruments were tested on the same day or with a minimum time delay, so the measurement conditions were kept constant. The test procedure for individual instruments was the same as for the first series of tests for determining the stability of errors over time.

**Figure 24:**
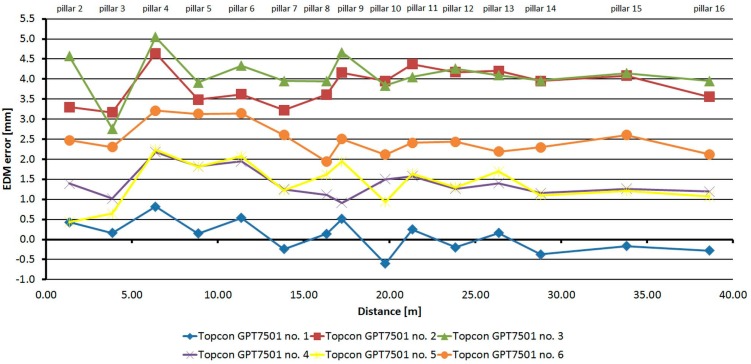
Comparison of absolute errors of six Topcon GPT-7501 instruments.

From Figure 24 it is clear that the development of errors of all six Topcon GPT-7501 instruments is similar (with only minor differences), but there is a difference in the size of the absolute errors, which is caused by the residual additive error. The difference of errors for these instruments reaches a maximum value of up to 5 mm.

**Figure 25:**
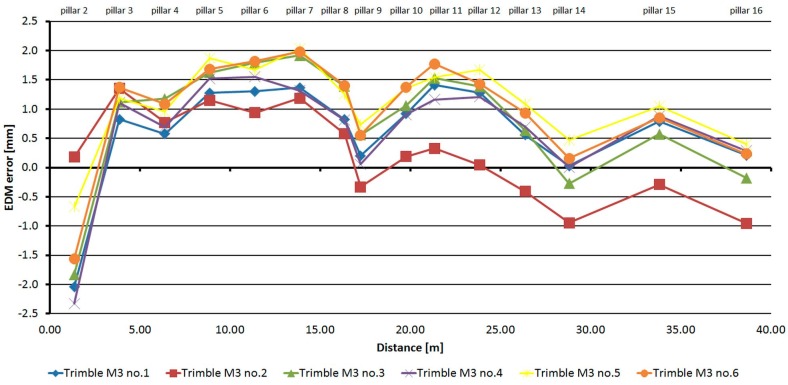
Comparison of absolute errors of six Trimble M3 instruments.

From Figure 25, it is clear that the development of errors of all six Trimble M3 instruments is similar (with only minor differences), but there is a difference in the size of absolute errors, which is caused by the residual additive error. The difference of errors for these instruments reaches a maximum value of up to 1.5 mm.

**Figure 26:**
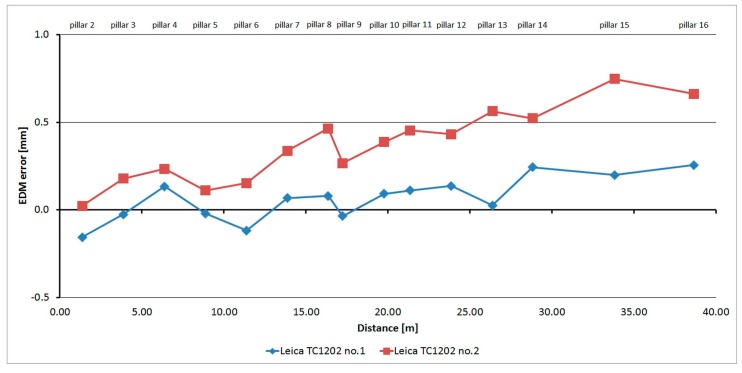
Comparison of absolute errors of two Leica TC1202 instruments.

**Figure 27:**
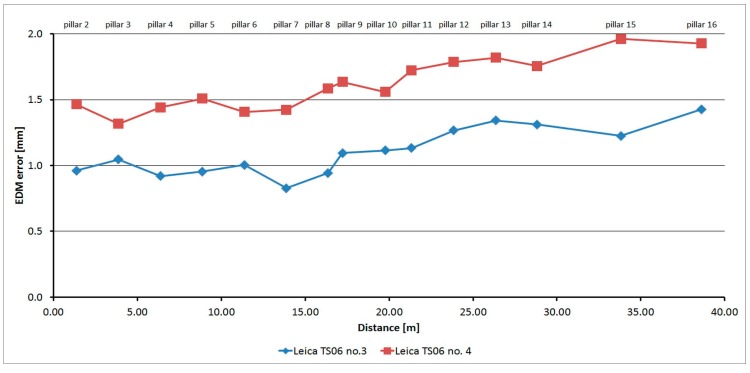
Comparison of absolute errors of two Leica TS06 instruments.

Comparing the Leica family, it appears that the development of absolute errors is similar and differs only in minor residual additive constants. For the Leica TC1202 instrument (Figure 26) the mean difference is 0.2 mm, for the Leica TS06 instrument (Figure 27) the mean difference is 0.5 mm. For both types it is obvious a gradual increase of size of the absolute errors occurs. This growth may be caused by the scale factor error or by the existence of a long periodic wave, which participates on the carrier wave of the distance meter.

Comparisons of instruments of the same type show that the absolute errors are similar, but differ in constant. This test confirmed the general rule that, for each instrument and the prism, a specific additive constant [8] must be determined and that if a correction function was created, it could not be used for a type of instrument (distance meter) but only for one particular instrument.

### 3.4. Determination of the Relative Errors of Measured Distances in Detail on the Baseline with an Interferometer

Based on the results of previous tests, four instruments were selected for detailed determination of their relative distance errors; namely, one Trimble S6, one Trimble M3, one Topcon GPT-7501 and one Leica TC1202. Measurements were performed in a metrology laboratory at a constant temperature of 19.5 °C.

**Figure 28:**
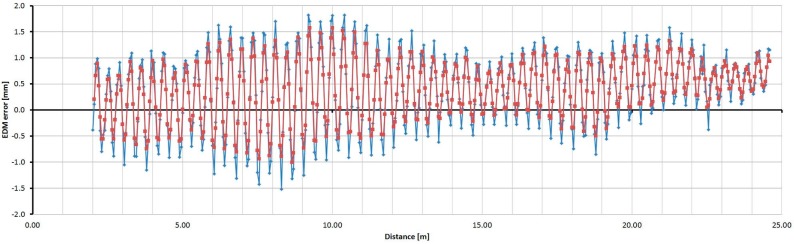
Relative errors of the Trimble S6 instrument (measured blue, averaged red).

For the Trimble S6 instrument, the basic step of testing was selected as 0.05 m to recognize the trend of the cyclic error, which has the basic unit length of 0.37 m. Figure 28 shows measured and averaged (by a moving average of the third grade) relative errors. The sizes of the errors are in the range of 3.5 mm. The cyclic error has a constant wavelength but variable amplitude; thus, the resulting error involves other harmonic influences.

**Figure 29:**
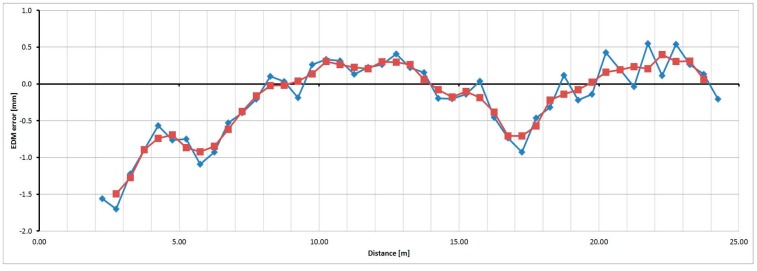
Relative errors of the Trimble M3 instrument (measured blue, averaged red).

For the Trimble M3 instrument the basic step of testing was selected as 0.50 m. Figure 29 shows measured and averaged (by a moving average of the third grade) relative errors. The sizes of the errors are in the range of 2.7 mm; their trend is approximately harmonic.

**Figure 30:**
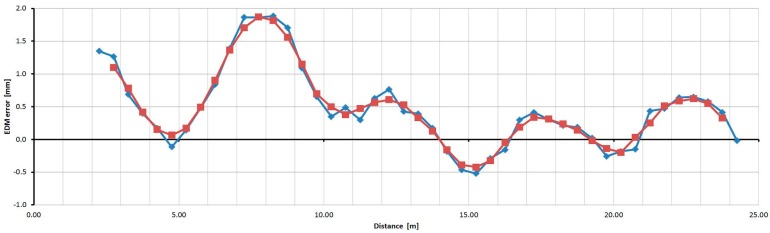
Relative errors of the Topcon GPT-7501 instrument (measured blue, averaged red).

For the Topcon GPT-7501 instrument, the basic step of testing was selected as 0.50 m. Figure 30 shows the measured and averaged (by a moving average of the third grade) relative errors. The sizes of the errors are in the range of 2.5 mm; their trend is approximately harmonic.

**Figure 31:**
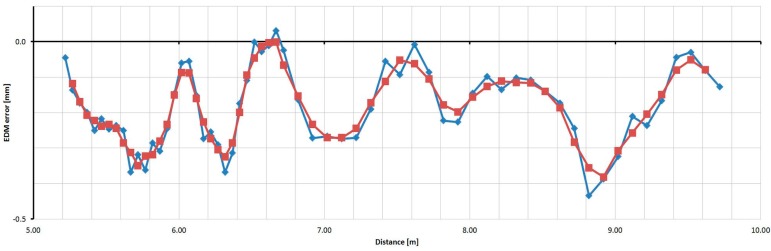
Relative errors of the Leica TC1202 instrument (measured blue, averaged red).

For the Leica TC1202 instrument, the basic step of testing was selected as 0.05 m and 0.1 m; A shorter interval of 4.5 m only was measured. Figure 31 shows the measured and averaged (by a moving average of the third grade) relative errors. The sizes of the errors are in the range of 0.4 mm. From the results of relative and absolute testing it is clear that the sizes of the errors are too small for the creation and successful use of the correcting function.

The testing revealed the range of relative errors which corresponds to the sizes observed in the tests of the repeated measurements. By combining the detailed trend of the relative errors and the absolute size of the errors of the instruments, a correction function could be created, the use of which should improve the accuracy of the measured distances.

### 3.5. The Resulting Trend of Errors for Three Selected Distance Meters

For each of the three tested instruments, average absolute errors were calculated from the data obtained by repeated measurements at the pillar baseline. Using these absolute values with the relative errors transformed (shifted) the result was the detailed trend of errors, which allowed the creation of the correction function.

**Figure 32:**
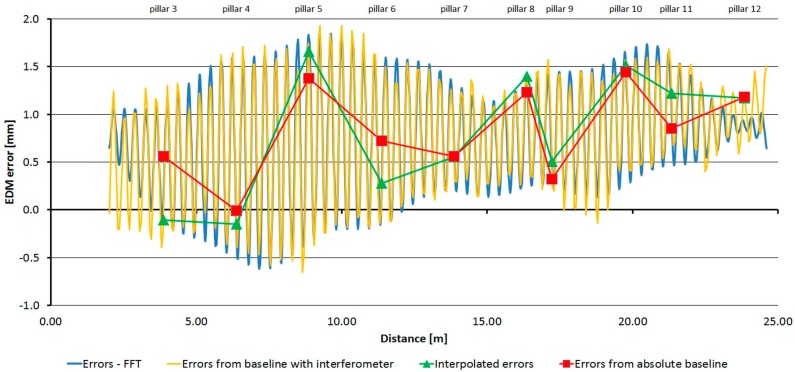
The resulting graph of the combination of absolute and relative errors and the correcting function by FFT–Trimble S6.

**Figure 33:**
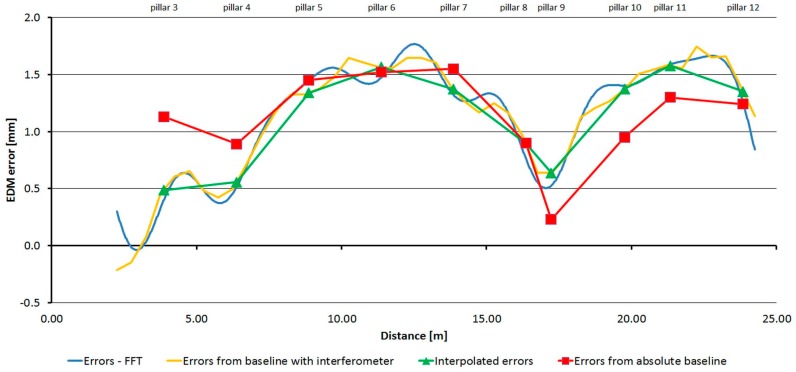
The resulting graph of the combination of absolute and relative errors and the correcting function by FFT–Trimble M3.

**Figure 34:**
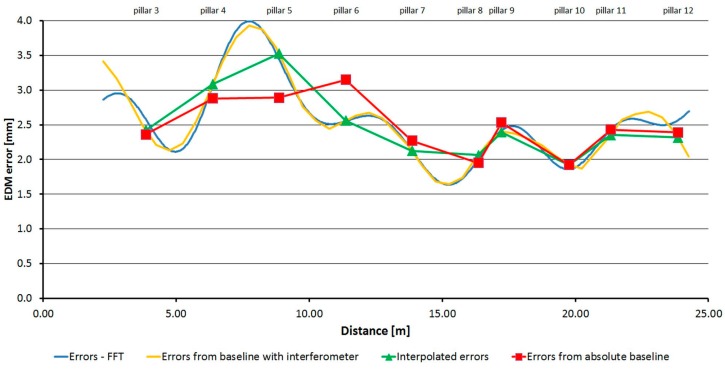
The resulting graph of the combination of absolute and relative errors and the correcting function by FFT–Topcon GPT-7501.

Figure 32, Figure 33 and Figure 34 show the detailed trends of errors determined by combining the data from the laboratory pillar baseline and the baseline with an interferometer. Errors determined by measurement at the pillar baseline are displayed in red and errors interpolated from the measurement at the baseline with an interferometer and transformed on the absolute ones are displayed in green. The shift was calculated by the robust (L_1_ norm) average to eliminate individual outliers. The orange line shows data from the baseline with an interferometer (transformed on the absolute data by the above mentioned shifts); from these data the corrections to the measured distances can be interpolated. The blue line shows a correction function created on the basis of the Fourier transformation application on the transformed relative errors (from the baseline with an interferometer). From those parameters determined by Fourier transform, only those whose amplitude was greater than 0.7 mm for the Trimble S6 instrument and greater than 1.0 mm for the Trimble M3 and Topcon GPT-7501 instruments were used.

The correction function for distance *d_k_* is given:

(11) $$correction_{k}=Y_{0}+\sum_{j=1}^{m}\left(A_{j}\cdot \sin\left(\frac{2\cdot \text{π}}{T_{j}}\cdot \left(d_{k}-d_{origin}\right)+{\text{ϕ}}_{j}\right)\right)$$

The parameters of the correction functions for each tested instrument are in Table 11, Table 12 and Table 13.

**Table 11:** Parameters of the correction function–Trimble S6.

Y_0_ = 0.7548 mm; d_origin_ = 2.001813 m		
Period T_j_ (m)	Amplitude A_j_ (mm)	Initial Phase φ_j_ (rad)
22.6500	0.1512	3.1605
11.3250	0.1361	3.1328
3.7750	0.0776	1.8739
0.3905	0.1145	−1.4533
0.3839	0.1812	1.5806
0.3775	0.5232	0.7886
0.3713	0.4352	4.4284
0.3653	0.1637	4.2012
0.3595	0.0860	3.8871

**Table 12:** Parameters of the correction function–Trimble M3.

Y_0_ = 1.1231 mm; d_origin_ = 2.250642 m		
Period T_j_ (m)	Amplitude A_j_ (mm)	Initial Phase φ_j_ (rad)
22.5000	0.3222	4.3280
11.2500	0.5167	3.2598
7.5000	0.2049	4.0981
4.5000	0.1380	4.0704
3.7500	0.1790	4.2301
2.8125	0.1208	3.3498

**Table 13:** Parameters of the correction function–Topcon GPT-7501.

Y_0_ = 2.5405 mm; d_origin_ = 2.249549 m		
Period T_j_ (m)	Amplitude A_j_ (mm)	Initial Phase φ_j_ (rad)
22.5000	0.5419	0.3230
11.2500	0.2630	4.1105
7.5000	0.2370	2.5339
5.6250	0.2872	2.1316
4.5000	0.2964	−0.1128
3.7500	0.1012	0.1919

### 3.6. Results of the Verification Experiment of the Correction Function’s Quality

**Figure 35:**
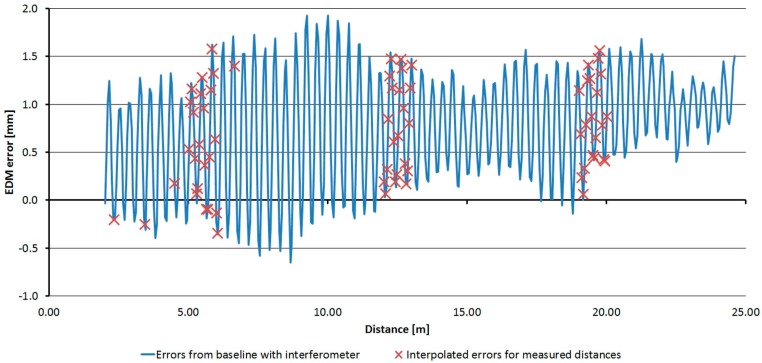
Interpolated corrections of the measured distances–Trimble S6.

**Figure 36:**
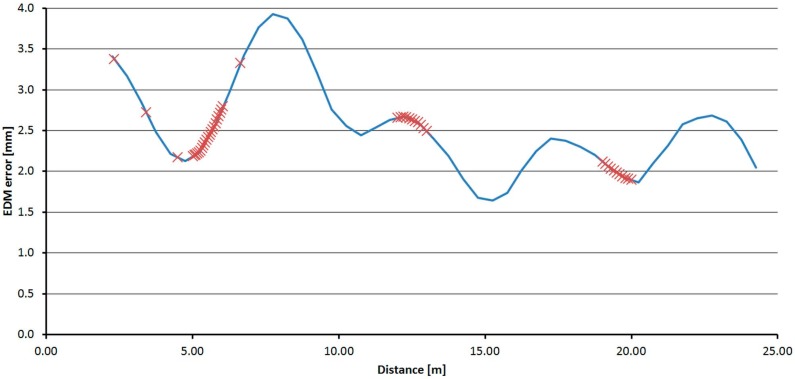
Interpolated corrections of the measured distances–Trimble M3.

**Figure 37:**
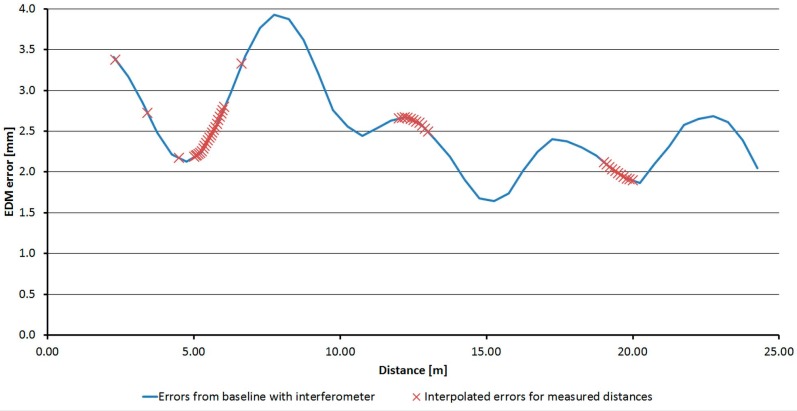
Interpolated corrections of the measured distances–Topcon GPT-7501.

**Figure 38:**
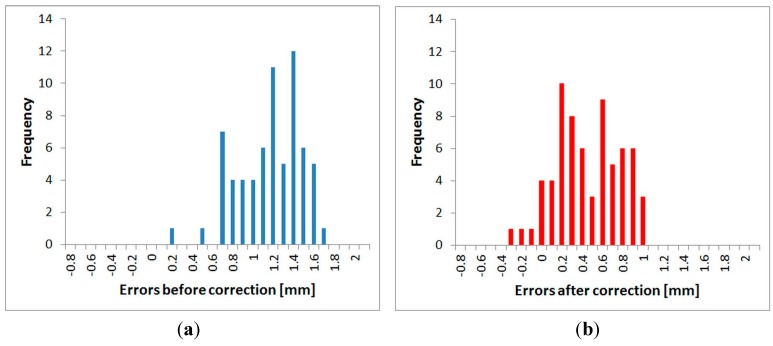
Distribution of the errors–Trimble S6: (**a**) before the correction; (**b**) after the correction.

**Figure 39:**
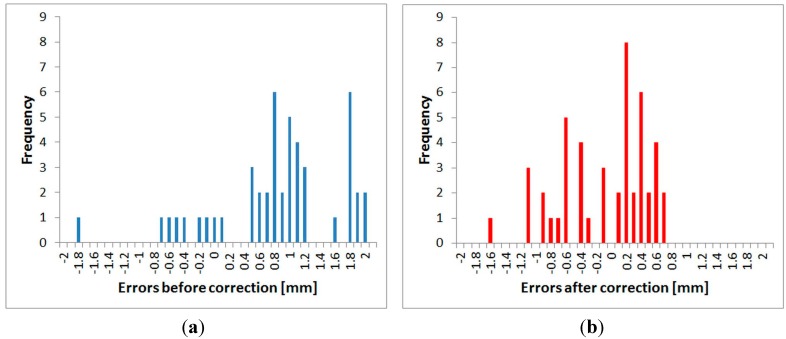
Distribution of the errors–Trimble M3: (**a**) before the correction; (**b**) after the correction.

**Figure 40:**
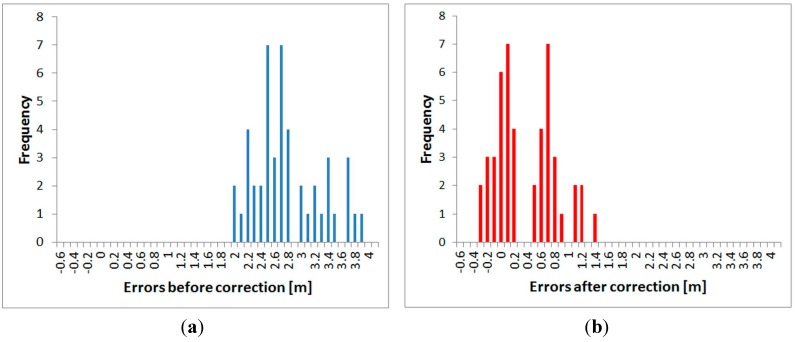
Distribution of the errors–Topcon GPT-7501: (**a**) before the correction; (**b**) after the correction.

All three fully-tested instruments were experimentally tested to determine the suitability of the application of the correction function. Distances directly measured by the tested instrument, and also distances corrected using the procedure described above, were compared with a reference distance measured by the Leica Absolute Tracker AT401. Measurements were performed in the laboratory with the pillar baseline at a stable temperature of 25.5 °C. For each instrument, absolute errors before and after the correction (both by interpolation and by the FFT correction function) were calculated from the differences of the reference and tested distances. The mean error and the standard deviation for each instrument were calculated from those values. The results are presented in Table 14, Table 15 and Table 16. Graphs (Figure 35, Figure 36 and Figure 37) were also created showing the measured distances and interpolated absolute errors (corrections). Histograms were also created showing the absolute errors before and after the application of interpolated corrections (Figure 38, Figure 39 and Figure 40).

**Table 14:** Effectiveness of the corrections application–Trimble S6, 67 distances.

Correction Method	Mean Error before Correction (mm)	Mean Error after Correction (mm)	Standard Deviation before Correction (mm)	Standard Deviation after Correction (mm)
Interpolation	1.117	0.400	1.158	0.511
Function (FFT)	1.117	0.386	1.158	0.556

**Table 15:** Effectiveness of the corrections application–Trimble M3, 47 distances.

Correction Method	Mean Error before Correction (mm)	Mean Error after Correction (mm)	Standard Deviation before Correction (mm)	Standard Deviation after Correction (mm)
Interpolation	0.800	−0.130	1.133	0.581
Function (FFT)	0.800	−0.161	1.133	0.631

**Table 16:** Effectiveness of the corrections application–Topcon GPT-7501, distances.

Correction Method	Mean Error before Correction (mm)	Mean Error after Correction (mm)	Standard Deviation before Correction (mm)	Standard Deviation after Correction (mm)
Interpolation	2.737	0.325	2.781	0.549
Function (FFT)	2.737	0.400	2.781	0.581

The histograms show that the implementation of the corrections moved the mean value of the errors closer to zero and that the resulting distribution is more similar to the normal distribution. Standard deviations characterizing the accuracy of the corrected distances are approximately 0.5 mm for all instruments. By comparing the standard deviations before and after correction, it can be observed that the accuracy is improved by more than 50% for all instruments (Table 14, Table 15 and Table 16). The results of the experiment confirmed the benefit of using the designed correction procedure for accurate distance measurements.

## 4. Conclusions

A procedure was designed for acquiring more precise and accurate results of distance measurements using EDM. This procedure is based on the suppression of systematic and random errors. The systematic errors are suppressed by corrections that depend on the measured distance. The corrections are determined on the base of a special correction procedure that uses both an absolute pillar baseline and a relative interferometric baseline. The random errors are suppressed by a measurement procedure modification that uses a large number of repetitions.

The proposed procedure of the testing and corrections application is original; during its design a huge number of experimental measurements were carried out. Namely, twenty instruments were tested, and more than 60,000 distances were measured. For the tests a new laboratory pillar baseline with forced centerings and an accuracy of the reference length of 0.02 mm was established. Instruments (distance meters of the total stations) were repeatedly tested on the baseline and it was discovered that, when a larger number of repetitions is used (16 or more were used), random errors were well-suppressed and only systematic errors remained. These systematic errors are almost constant under similar conditions. By comparing instruments of the same type, it was shown that the same distance meters have nearly the same errors, but differ significantly in the additive constant.

In the case of the Leica instruments, it was found that the variations of the absolute errors are very small and, therefore, further implementation of a correction function is irrelevant; only the additive constant needed to be adjusted. In the case of the Trimble and Topcon instruments, the absolute errors exceeded 1 mm and creation of a correction function seemed to be relevant and useful. For three instruments the detailed trend of the relative errors was then determined, using the baseline with an interferometer; the errors were then transformed to absolute ones (using absolute errors determined on the laboratory pillar baseline) and on this basis corrective procedures were determined. The usefulness of the designed procedure was experimentally proven. A standard deviation of 0.5 mm was achieved for all three tested instruments, leading to lowering of the standard deviation by 50%.

This procedure is suitable and can be used mainly in industrial engineering surveying, where the measurement takes place in closed halls and over short distances. For each instrument it is possible to create a correction function (for a specific distance interval) and then apply that correction to multiple measured distances, thereby improving the quality of the results. The authors suggest that further research will focus on the effect of temperature changes on the size and trend of the systematic errors.
